# Anatomic Landmarks for Basal Joint Injections

**Published:** 2012-01-18

**Authors:** Ron Hazani, Nitin J. Engineer, Josh Elston, Bradon J. Wilhelmi

**Affiliations:** University of Louisville School of Medicine, Division of Plastic surgery, Louisville, KY

## Abstract

**Objective:** Basal joint arthritis is a common cause of pain and disability, particularly in elderly women. Corticosteroid injection with splinting provides a reliable long-term relief for patients with mild arthritis. Proper location of the basal joint with anatomic landmarks can facilitate diagnosis and treatment of basal joint arthritis while avoiding inadvertent injury to local structures. The purpose of this study is to identify bony anatomic landmarks for basal joint injections and aid clinicians in avoiding inadvertent injury to surrounding structures on the radial side of the wrist. **Methods:** Twenty fresh cadaveric wrists were dissected with the aid of loupe magnification. The distal edge of the radial styloid and the palpable dorsal aspect of the thumb metacarpophalangeal joint were used as bony anatomic landmarks for the identification of the basal joint along a longitudinal vector. Measurements of the distance from our anatomic landmarks to the basal joint space were recorded. The locations of the radial artery and the superficial branch of the radial nerve were noted in relation to the borders of the anatomic snuffbox at the basal joint level. **Results:** The basal joint of the thumb is located 2.44 ± 0.34 cm distal to the distal edge of the radial styloid, and 4.47 ± 0.29 cm proximal to the metacarpophalangeal joint. At the level of the basal joint, the radial artery is found 0.76 ± 0.12 cm dorsal to the extensor pollicis brevis tendon. The first branch of the superficial branch of the radial nerve is volar to the abductor pollicis longus tendon in 84% of the specimens and courses over the abductor pollicis longus tendon in 16%. **Conclusion:** The basal joint of the thumb is approximately 2.4 cm distal to the radial styloid and 4.5 cm proximal to the metacarpophalangeal joint. Placement of a needle in the basal joint space immediately dorsal to extensor pollicis brevis tendon while applying longitudinal traction on the thumb is more likely to avoid damage to the radial artery and the superficial branch of the radial nerve.

The basal joint allows for the greatest freedom of motion in the hand. It is responsible for placing the thumb in innumerable positions and its stability allows for a fine and strong pinch.^1^ The basal joint is the second most commonly involved site of osteoarthritis after the distal interphalangeal joint.^2^ Nonetheless, its involvement causes far more significant functional disability secondary to pain and weakness.^2^

During the early stages of basal joint arthritis, steroid injections with splinting can provide a reliable long-term relief.^3^ Accuracy of needle placement has generated much controversy in the literature regarding the need for image-guided location of the joint, and may depend on the experience of the practicing clinician.^4^^-^7 In most cases, the palpable radial and proximal edge of the base of the first metacarpal can be used as a reliable landmark. Occasionally, a patient may present with a “thicker” wrist, which can be a challenge to the inexperienced examining clinician. Finding the basal joint in these cases is particularly difficult as the joint is nestled at the base of the anatomic snuffbox. Superficial bony anatomic landmarks for the basal joint can facilitate a safer and more reliable approach to the percutaneous treatment of this disease. In our online PubMed literature search, no previous anatomic landmark studies for the basal joint were found. The purpose of this study is to identify bony anatomic landmarks for basal joint injections and aid clinicians in avoiding inadvertent injury to the surrounding structures of the radial wrist.

## METHODS

**Figure 1:**
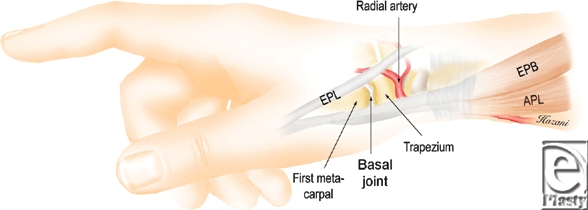
The basal joint within the anatomic snuffbox is bounded volarly by the tendons of the abductor pollicis longus (APL) and extensor pollicis brevis (EPB), and dorsally by the tendon of the extensor pollicis longus (EPL).

The basal joint is nestled within the anatomic snuffbox at the radial aspect of the wrist. The boundaries of the three-sided snuffbox are easily palpable by placing the thumb in a fully abducted position. It is bounded on the palmar side by the tendons of the abductor pollicis longus (APL) and extensor pollicis brevis (EPB), and dorsally by the tendon of the extensor pollicis longus (EPL). The distal edge of the radial styloid forms the proximal border. The apex of the schematic snuffbox isosceles triangle forms the distal border (Fig 1).^8^

**Figure 2:**
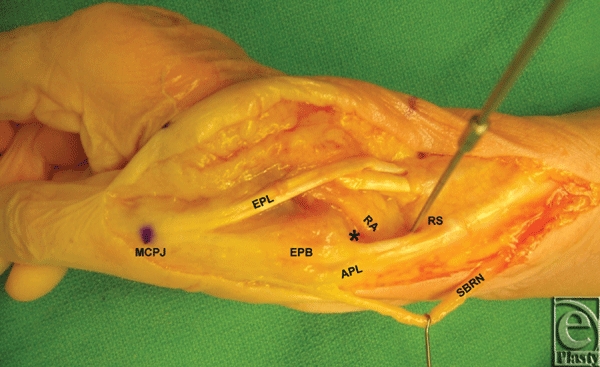
A photo demonstrating one cadaveric specimen following dissection of the radial wrist, depicting the boundaries of the anatomic snuffbox, the basal joint, and its bony anatomic landmarks.* indicates basal joint space; APL, Abductor pollicis longus; EPB, extensor pollicis brevis; EPL, extensor pollicis longus; MCPJ, metacarpophalangeal joint; RA, radial artery; RS, wire placed at the distal edge of the radial styloid; SBRN, superficial branch of the radial nerve.

Twenty fresh cadaveric wrists were dissected with the aid of loupe magnification. The distal edge of the radial styloid (RS) and the palpable dorsal aspect of the thumb metacarpophalangeal (MCP) joint were used as bony anatomic landmarks for predicting the location of the basal joint along a longitudinal vector. For consistent measurements from the basal joint to its anatomic landmarks, the wrist and thumb were placed in the following position. The wrist was at 30° of extension with no radial or ulnar deviation. The thumb was at a rest position situated in a plane at about a 60° angle to that of the palm and its palmar surface was facing ulnarly. The metacarpophalangeal (MCP) joint was at 30° of flexion and the interphalangeal (IP) joint was at 45° of flexion. All structures were then aligned along the longitudinal axis of radial wrist. A longitudinal incision was created, extending from the radial aspect of the distal forearm to the midline of the thumb dorsal aspect of the IP joint. Skin and subcutaneous dissection facilitated exposure of the snuffbox borders, the basal joint, the superficial branch of the radial nerve (SBRN), and the radial artery (Fig 2). Measurements of the distance from the basal joint to our anatomic landmarks (mean ± standard deviation), the course of the SBRN and location radial artery within the snuffbox were recorded.

## RESULTS

**Figure 3:**
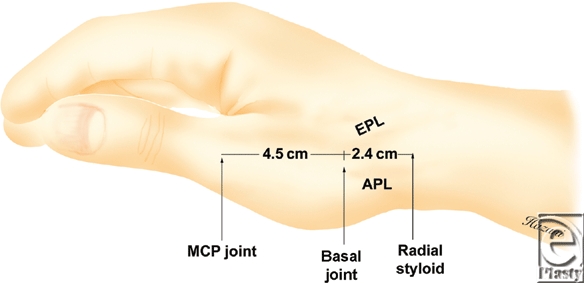
The distal edge of the radial styloid and the palpable dorsal metacarpophalangeal (MCP) joint as bony anatomic landmarks for predicting the location of the basal joint along a longitudinal vector. The basal joint is approximately 2.4 cm distal to the radial styloid and 4.5 cm proximal to the MCP joint.

The basal joint of the thumb is located 2.44 ± 0.34 cm distal to the distal edge of the RS, and 4.47 ± 0.29 cm proximal to the MCP joint (Fig 3). At the level of the basal joint space, the radial artery is found 0.76 ± 0.12 cm dorsal to the EPB tendon. The first branch of the SBRN is volar to the APL tendon in 84% of the specimens and coursing over the APL tendon in 16%. In all of the specimens, the second branch of the SBRN and its smaller contributions, course over the distal aspect of the snuffbox—away from the basal joint space.

## DISCUSSION

Intra-articular corticosteroid injections have been used since the 1950s to alleviate pain and swelling in arthritic joints.^9^ In a prospective study of 30 patients, Day et al^3^ demonstrated that steroid injection with splinting provided reliable long-term relief in thumbs with Eaton stage 1 disease. All of the injections were administered by the senior hand surgeons and without the aid of fluoroscopy. It is important to recognize that the method of injection can vary significantly and may depend on the clinician's level of training. Some authors advocate the use of fluoroscopy or ultrasound as a guide for proper intra-articular needle placement. Pollard et al^6^ reported a 100% rate of intra-articular accuracy with fluoroscopy-guided injections as compared with 82% in the traditional “blind” group. In an ultrasound-guided cadaveric study, injections to the trapeziometacarpal joint were successful in 94% of the specimens.^5^

Advocates of the traditional “blind” injection technique (one that does not rely on ultrasound or fluoroscopy) claim that the use of anatomic landmarks is a reliable method and probably obviates the need for radiologic guidance.^7^ No specific landmarks were discussed in their study. In this clinical study of 32 symptomatic patients, ultrasound evidence of intra-articular injection was demonstrated in all patients.^7^ Mandl et al^7^ suggest that contrasting findings with regard to the practice of “blind” injections in the office may require an experienced clinician.

As an adjunct to clinical experience, we suggest the use of the RS and the MCP joint of the thumb as reliable superficial anatomic landmarks for the basal joint. Soft tissue landmarks can be difficult to identify in “thicker” wrists and possibly change with motion. Therefore, the use of easily palpable bony landmarks such as the RS and MCP joint is likely to be superior to other structures when palpating the radial wrist structures.

In addition to locating the basal joint at the radial wrist, predicting the course of neurovascular structures traversing the anatomical snuffbox is imperative. Although there are no reports of injured SBRN and radial artery for basal joint injections, it is necessary to recognize the location of such structures at the level of the basal joint. Inadvertent injury to the SBRN can cause pain, numbness, and dysesthesias. Injury to the radial artery may result in bleeding or a false aneurysm.^10^ This jeopardy is hypothetical in view of the fact that there have been no previously reported cases. We relate to the results of Steinberg et al^10^ regarding a safe zone within the proximal anatomic snuffbox and propose an additional window of safety at the level of the basal joint.

Using our longitudinal measurements from the radial styloid and the dorsal aspect of the thumb MCP joint, a needle can safely be inserted immediately dorsal to the EPB tendon. In this location, it is less likely to injure the radial artery as it courses approximately 7 mm dorsal to the EPB tendon. In addition, the first branch of the SBRN is safely located volar to the EPB tendon. While needle placement immediately dorsal to the APL tendon is safe, the EPB tendon is the most dorsal structure of the two tendons, which is easier to identify within the boundaries of the snuffbox (Fig 2). Longitudinal traction on the thumb will widen the basal joint space distal to the radial artery and avoid puncture of the traversing artery. We recommend caution with any needle advancement causing sharp pain, numbness, or tingling—indicating injury to the first branch due to an anomalous location or to cutaneous nerves branching off of the second branch of the SBRN. Although not encountered in our dissections, the lateral cutaneous nerve of the forearm may also innervate the radial aspect of the thumb in an anomalous distribution.^11^

In conclusion, anatomic landmarks for the basal joint can facilitate a safe and reliable approach to the percutaneous treatment of basal joint arthritis. The basal joint is approximately 2.4 cm distal to the RS and 4.5 cm proximal to the MCP joint. Placement of a needle immediately dorsal to the EPB tendon while applying longitudinal traction on the thumb is less likely to cause damage to the radial artery and the superficial branch of the radial nerve.
